# Prediction of Methotrexate Intolerance in Juvenile Idiopathic Arthritis: a prospective, observational cohort study

**DOI:** 10.1186/s12969-015-0002-3

**Published:** 2015-02-18

**Authors:** Evert Hendrik Pieter van Dijkhuizen, Maja Bulatović Ćalasan, Saskia MF Pluijm, Maurits CFJ de Rotte, Sebastiaan J Vastert, Sylvia Kamphuis, Robert de Jonge, Nico M Wulffraat

**Affiliations:** Department of Paediatric Immunology, University Medical Centre Utrecht, Wilhelmina Children’s Hospital, Utrecht, The Netherlands; Department of Paediatric Haemato-Oncology, Erasmus University Medical Centre Rotterdam, Sophia Children’s Hospital, Rotterdam, The Netherlands; Department of Clinical Chemistry, Erasmus University Medical Centre Rotterdam, Rotterdam, The Netherlands; Department of Paediatric Rheumatology, Erasmus University Medical Centre Rotterdam, Sophia Children’s Hospital, Rotterdam, The Netherlands; Pediatria II, Reumatologia, IRCCS G. Gaslini, Largo Gaslini 5, 16147 Genova, Italy

**Keywords:** Juvenile idiopathic arthritis, Methotrexate, Adverse events, Methotrexate intolerance, Prediction model, Predictor

## Abstract

**Background:**

Methotrexate (MTX) is an effective and safe drug in the treatment of juvenile idiopathic arthritis (JIA). Despite its safety, MTX-related gastrointestinal adverse effects before and after MTX administration, termed MTX intolerance, occur frequently, leading to non-compliance and potentially premature MTX termination. The aim of this study was to construct a risk model to predict MTX intolerance.

**Methods:**

In a prospective JIA cohort, clinical variables and single nucleotide polymorphisms were determined at MTX start. The Methotrexate Intolerance Severity Score was employed to measure MTX intolerance in the first year of treatment. MTX intolerance was most prevalent at 6 or 12 months after MTX start, which was defined as the outcome for the prediction model. The model was developed in 152 patients using multivariable logistic regression analysis and subsequently internally validated using bootstrapping.

**Results:**

The prediction model included the following predictors: JIA category, antinuclear antibody, parent/patient assessment of pain, Juvenile Arthritis Disease Activity Score-27, thrombocytes, alanine aminotransferase and creatinine. The model classified 77.5% of patients correctly, and 66.7% of patients after internal validation by bootstrapping. The lowest predicted risk of MTX intolerance was 18.9% and the highest predicted risk was 85.9%. The prediction model was transformed into a risk score (range 0–17). At a cut-off of ≥6, sensitivity was 82.0%, specificity 56.1%, positive predictive value was 58.7% and negative predictive value 80.4%.

**Conclusions:**

This clinical prediction model showed moderate predictive power to detect MTX intolerance. To develop into a clinically usable tool, it should be validated in an independent cohort and updated with new predictors. Such an easy-to-use tool could then assist clinicians in identifying patients at risk to develop MTX intolerance, and in turn to monitor them closely and intervene timely in order to prevent the development of MTX intolerance.

**Trial registration:**

ISRCTN register, www.isrctn.com, ISRCTN13524271

## Background

Juvenile idiopathic arthritis (JIA) is the most common childhood rheumatic disease [1,2]. In JIA, methotrexate (MTX) is the cornerstone treatment, due to its efficacy and safety. Serious adverse effects such as hepatotoxicity and bone marrow suppression occur rarely [3]. In contrast, MTX-related gastrointestinal adverse effects, such as nausea, abdominal pain and vomiting, occur frequently [4-10]. Folic acid supplementation is an accepted strategy to prevent and treat these adverse effects [11-13]. Despite folic acid use, many JIA patients experience gastrointestinal adverse effects after MTX intake [4-10]. JIA patients also experience anticipatory adverse effects, occurring before MTX administration (at the sight of MTX), and associative adverse effects, occurring when thinking of MTX administration (its colour or smell) [4,5,14]. These adverse effects are thought to be a result of classical conditioning to the abovementioned physical symptoms experienced after MTX intake [14]. Importantly, if physical symptoms are absent, conditioned responses cannot develop [15]. Such a combination of symptoms, which we previously termed MTX intolerance, [14] is a significant burden for JIA patients and their parents. Notably, MTX intolerance occurs in up to half of JIA patients on MTX, [14] and can negatively affect their quality of life [6]. Moreover, over three-quarters of intolerant patients reluctantly used or even refused MTX, [14] which, besides leading to non-compliance, could lead to premature discontinuation of MTX, and even replacement by costly biologicals [5,16,17]. Such consequences could be avoided, if the development of MTX intolerance is prevented.

To prevent MTX intolerance, it is crucial to predict which patients starting MTX will be at risk to develop it. Thus, clinicians could be able to prevent MTX intolerance in patients at risk by immediate treatment of emerging physical symptoms, which otherwise could give rise to conditioned responses. Treatment of physical symptoms could include lowering the MTX dose, [4] or starting behavioural therapy [5] or anti-emetics [18]. Predicting MTX intolerance would enable clinicians to apply such treatment strategies only in those patients who are likely to develop MTX intolerance.

Single nucleotide polymorphisms (SNPs) involved in the MTX metabolic pathways, and clinical predictors have been associated with MTX-related gastrointestinal adverse effects in rheumatoid arthritis (RA) [19-28] and JIA, the latter of which were reviewed recently [29]. However, to date no model has been constructed to predict MTX intolerance in JIA. The aim of this cohort study was to develop and internally validate such a prediction model, using clinical and genetic predictors.

## Methods

### Patients and study design

An investigator-initiated observational prospective study on efficacy and adverse effects of MTX in patients starting MTX (ISRCTN13524271) was performed at the University Medical Centre Utrecht and Erasmus University Medical Centre Rotterdam, The Netherlands, between January 2008 and October 2012. It was approved by the Ethics Committees of the participating centres and the Central Committee on Research involving Human Subjects, and was conducted according to good clinical practice guidelines.

Patients aged 1–18 years, with a confirmed diagnosis of JIA according to International League of Associations for Rheumatology (ILAR) criteria, [30] who started MTX, were included. Those who had stopped MTX for at least three months, but re-started MTX due to a relapse, were also included. At the time of MTX start, their clinical data (Table 1) were documented in case report forms and blood for the analysis of SNPs was drawn.

**Table 1:** **Prevalence, univariable ORs (95%-CI) and p-values for potential predictors of MTX intolerance at MTX start**

		**Cohort, n = 152**		
**Variables**		**Frequency n (%)** ^**a**^	**OR (95%-CI)**	**p-value**
*Demographics*				
Female		92 (60.5)	1.34 (0.64-2.82)	0.432
Age at disease onset	>8 years	80 (52.6)	0.68 (0.34-1.36)	0.271
Age at MTX start^*^	>12 years	72 (47.4)	0.54 (0.27-1.07)	0.073
Disease duration at MTX start	>0.5 years	103 (67.8)	0.79 (0.37-1.70)	0.535
*JIA category* ^*b^				
Oligoarticular (persistent/extended)		62 (40.8)	Reference	0.094
Polyarticular (RF negative/positive)		64 (42.1)	1.91 (0.86-4.24)	
Other (systemic/psoriatic/enthesitis)		26 (17.1)	0.78 (0.27-2.31)	
*Disease characteristics*				
ANA^*b,c^	Positive	84 (55.3)	1.98 (0.97-4.07)	0.057
RF^**c**^	Positive	16 (10.5)	1.52 (0.62-3.72)	0.352
HLA-B27^**c**^	Positive	11 (7.2)	0.78 (0.29-2.12)	0.510
Uveitis	Present	21 (13.8)	1.44 (0.55-3.78)	0.455
*Disease activity*				
CHAQ disability score^**c**^	≤0.250	36 (23.7)	Reference	0.395
	0.250-1.875	88 (57.9)	0.61 (0.24-1.55)	
	>1.875	15 (9.9)	0.72 (0.18-2.80)	
Parent/patient assessment of pain^*b,c^	≤3 cm	58 (38.2)	Reference	0.086
	3-6 cm	36 (23.7)	2.19 (0.84-5.67)	
	>6 cm	42 (27.6)	0.78 (0.30-2.02)	
Parent/patient global assessment^**c**^	>2.5 cm	90 (59.2)	0.79 (0.36-1.72)	0.494
Active joints^*^	>2	92 (60.5)	2.00 (0.91-4.41)	0.070
Limited joints^*^	>1	108 (71.1)	2.02 (0.92-4.46)	0.072
PGA^**d**^	≤2 cm	50 (32.9)	Reference	0.496
	2-5 cm	86 (56.6)	1.35 (0.53-3.47)	
	>5 cm	16 (10.5)	0.87 (0.21-3.60)	
ESR^**c**^	>15 mm/hr	74 (48.7)	1.46 (0.66-3.25)	0.341
CRP^**c**^	>10 mg/L	49 (32.2)	0.83 (0.40-1.74)	0.544
JADAS-27^*b,c^	≤5	16 (10.5)	Reference	0.048
	5-15	59 (38.8)	0.40 (0.11-1.40)	
	>15	52 (34.2)	0.93 (0.25-3.44)	
*Biochemical variables* ^***c***^				
Haemoglobin	>7.5 mmol/L	78 (51.3)	1.18 (0.60-2.32)	0.620
Leucocytes	>7 × 10^9^/L	96 (63.2)	1.21 (0.59-2.47)	0.606
Thrombocytes^*b^	>350 × 10^9^/L	74 (48.7)	1.61 (0.82-3.16)	0.161
AST	>17 IU/L	96 (63.2)	1.08 (0.50-2.36)	0.635
ALT^*b^	>12 IU/L	101 (66.4)	0.41 (0.19-0.88)	0.019
Creatinine^*b^	>50 μmol/L	56 (36.8)	0.51 (0.24-1.08)	0.069
*Medication*				
MTX dose, median (IQR)	mg/m^2^/week	9.9 (9.0-11.2)	NA	
MTX route	oral	148 (97.4)	NA	
MTX restarted		31 (20.4)	1.22 (0.48-3.11)	0.554
Folic acid		150 (98.7)	NA	
Anti-emetics		5 (3.3)	NA	
NSAID		120 (78.9)	0.93 (0.38-2.28)	0.655
*Single nucleotide polymorphisms* ^***c***^				
*MTHFR* rs1801133 C > T	TT	15 (9.9)	0.60 (0.21-1.69)	0.322
*MTHFR* rs1801131 A > C	CC/AC	79 (52.0)	1.65 (0.76-3.62)	0.201
*MTRR* rs1801394 A > G^*^	GG/AG	117 (77.0)	0.53 (0.24-1.20)	0.123
*RFC/SLC19A1* rs1051266 C > T^*^	TT	17 (11.2)	1.77 (0.74-4.25)	0.194
*ITPA* rs1127354 C > A	AA/CA	15 (9.9)	0.62 (0.22-1.74)	0.350
*AMPD1* rs17602729 G > A	AA/GA	41 (27.0)	1.46 (0.70-3.05)	0.304
*ATIC* rs2372536 C > G	GG/CG	93 (61.2)	0.84 (0.39-1.83)	0.614
*ADA22* rs73598374 C > T	TT/CT	13 (8.6)	NA	
*ADORA2A* rs5751876 C > T	TT	28 (18.4)	1.54 (0.65-3.64)	0.319
*MDR-1/ABCB1* rs 128503 G > A^*^	AA	32 (21.1)	1.73 (0.75-3.98)	0.190
*MDR-1/ABCB1* rs1045642 G > A	AA	44 (28.9)	1.40 (0.65-3.01)	0.376
*MDR-1/ABCB1* rs2032582 C > A/T	AA/TT	24 (15.8)	1.51 (0.63-3.64)	0.344
*MRP-1/ABCC1* rs35592 T > C	CC/TC	52 (34.2)	0.79 (0.39-1.57)	0.494
*MRP-1/ABCC1* rs3784862 A > G	GG/AG	73 (48.0)	0.97 (0.50-1.91)	0.824
*MRP-2/ABCC2* rs4148396 C > T	TT	18 (11.8)	1.57 (0.60-4.08)	0.349
*MRP-2/ABCC2* rs717620 C > T	TT/CT	44 (28.9)	0.82 (0.37-1.82)	0.626
*MRP-3/ABCC3* rs4793665 T > C	CC/TC	92 (60.5)	0.73 (0.36-1.49)	0.381
*MRP-3/ABCC3* rs3785911 A > C^*^	CC/AC	78 (51.3)	1.67 (0.84-3.32)	0.136
*MRP-4/ABCC4* rs868853 T > C	CC/TC	22 (14.5)	0.88 (0.35-2.18)	0.734
*MRP-4/ABCC4* rs2274407 C > A	AA/CA	20 (13.2)	1.33 (0.48-3.73)	0.514
*MRP-5/ABCC5* rs2139560 G > A	AA/GA	92 (60.5)	1.31 (0.64-2.68)	0.450
*BCRP/ABCG2* rs13120400 T > C	CC/TC	63 (41.4)	0.77 (0.38-1.59)	0.470
*BCRP/ABCG2* rs2231142 G > T	TT/GT	30 (19.7)	0.96 (0.42-2.20)	0.744
*FPGS* rs4451422 A > C	CC/AC	102 (67.1)	1.37 (0.63-2.94)	0.417
*GGH* rs10106587 A > C	CC/AC	73 (48.0)	1.20 (0.59-2.46)	0.508
*GGH* rs3758149 G > A	AA/GA	77 (50.7)	1.20 (0.57-2.55)	0.602
*PCFT/SLC46A1* rs2239907 C > T	TT/CT	104 (68.4)	1.49 (0.69-3.23)	0.306

***Abbreviations:*** ALT, alanine aminotransferase; ANA, antinuclear antibody; AST, asparagine aminotransferase; CHAQ, childhood health assessment questionnaire; CI, confidence interval; CRP, C-reactive protein; ESR, erythrocyte sedimentation rate; HLA, human leucocyte antigen; IQR, interquartile range; IU, international units; JADAS, juvenile arthritis disease activity score; JIA, juvenile idiopathic arthritis; MICE, multivariate imputation by chained equations; MTX, methotrexate; NSAID, non-steroidal anti-inflammatory drug; OR, odds ratio; PGA, physician global assessment; RF, rheumatoid factor.

*Variables associated with the outcome at p < 0.20 in the univariable logistic regression analysis. Variables with observed frequencies of <5 in the cross-tabulation with the outcome were excluded from the univariable logistic analysis: MTX route, use of folic acid, use of anti-emetics and *ADA22* rs73598374*.*

^**a**^Frequencies are based on observed data, not imputed data.

^**b**^JIA category, ANA, parent/patient assessment of pain, JADAS-27, thrombocytes, ALT and creatinine were included in the multivariable logistic regression analysis.

^**c**^MICE was used to impute missing values in the following variables (percentage of missing values): HLA-B27 (60.5), RF (19.1), JADAS-27 (16.4), CRP (15.8), parent/patient global assessment (11.8), *RFC/SLC19A1* rs1051266 (11.8), creatinine (11.2), parent/patient assessment of pain (10.5), CHAQ disability score (8.6), *MDR-1/ABCB1* rs2032582 (8.6), ALT (7.9), AST (7.2), ESR (5.3), *GGH* rs3758149 (4.6), *MRP-2/ABCC2* rs717620 (3.9), *MRP-4/ABCC4* rs868853 (3.9), *MRP-5/ABCC5* rs2139560 (3.9), *GGH* rs10106587 (3.9), *MTHFR* rs1801131 (3.3), *ATIC* rs2372536 (3.3), *ADORA2A* rs5751876 (3.3), *MRP-1/ABCC1* rs3784862 (3.3), *MRP-2/ABCC2* rs4148396 (3.3), *MRP-3/ABCC3* rs4793665 (3.3), *BCRP/ABCG2* rs13120400 (3.3), *PCFT/SLC46A1* rs2239907 (3.3), *MTHFR* rs1801133 (2.6), *MTRR* rs1801394 (2.6), *ITPA* rs1127354 (2.6), *AMPD1* rs17602729 (2.6), *ADA22* rs73598374 (2.6), *MDR-1/ABCB1* rs1128503 and rs1045642 (2.6), *MRP-1/ABCC1* rs35592 (2.6), *MRP-3/ABCC3* rs3785911 (2.6), *MRP-4/ABCC4* rs2274407 (2.6), *BCRP/ABCG2* rs2231142 (2.6), *FPGS* rs4451422 (2.6), thrombocytes (2.0), ANA (2.0), hemoglobin (1.3), leucocytes (1.3).

^**d**^PGA was determined retrospectively by an experienced physician (SJV) in 20 visits (13.2%).

All patients completed the previously developed and validated MTX Intolerance Severity Score (MISS) at 3, 6 and 12 months after MTX start [14]. This questionnaire consists of 12 questions, assessing abdominal pain, nausea and vomiting after or before (anticipatory) MTX intake and when thinking of MTX (associative). Furthermore, it assesses behavioural complaints associated with MTX intake, such as crying, restlessness, irritability and refusal to take the drug. The score ranges from 0 to 36 and those with a score of ≥6, including at least one anticipatory, associative or behavioural symptom, were defined as MTX intolerant [14].

### Development of MTX intolerance over time and patient selection

**Figure 1:**
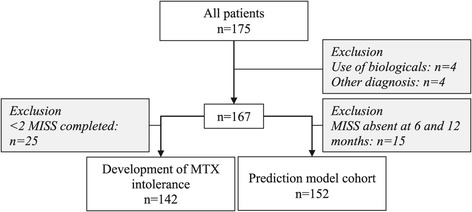
**Flowchart.** Abbreviations: MISS, Methotrexate Intolerance Severity Score; MTX, methotrexate.

To define the outcome for the prediction model, the development of MTX intolerance at 3, 6 and 12 months after MTX start was assessed. For this analysis, of 175 patients starting MTX treatment, 8 patients were excluded due to a diagnosis other than JIA (n = 4: Lyme disease, colitis, sarcoidosis, 22q11 deletion syndrome) and use of biologicals at MTX start (n = 3: anakinra; n = 1: etanercept), resulting in 167 eligible patients (Figure 1). Additionally, 25 patients who completed only one MISS during follow-up were excluded, as their development of MTX intolerance could not be determined. Therefore, the development of MTX intolerance was assessed in 142 patients (Figure 1). In the first year after MTX start, 59 (41.5%) patients were intolerant (score ≥6 with at least one anticipatory, associative or behavioural complaint) (Table 2). At 3 months, 22 (15.7%) patients were intolerant. However, intolerance resolved in the majority of these (13 [59.1%]) at 6 months. At 6 months, the number of intolerant patients increased to 33 (24.1%), of whom 24 (72.7%) were newly intolerant. At 12 months, 14 (42.4%) of those intolerant at 6 months stayed intolerant, whereas 8 had less than 6 points on the MISS and 11 did not complete it. The total number of intolerant patients at 12 months was 30 (23.3%), of whom 13 (43.3%) were newly intolerant (Table 2).

**Table 2:** **MTX intolerance development**

**Time point**	***N***	**Intolerance, n(%)** ^**a**^
3 months	140^**b**^	22 (15.7)
6 months	137^**b**^	33 (24.1)
12 months	129^**b**^	30 (23.3)
First treatment year	142	59 (41.5)
6 or 12 months^**d**^	152^**c**^	51 (33.6)

***Abbreviations:*** MTX, methotrexate; n, number of patients.

^**a**^Frequencies are based on observed data_;_^**b**^Patients still on MTX; ^**c**^Cohort for prediction model construction; ^**d**^Outcome was imputed in 21.7% of cases.

Taken together, the majority of patients developing MTX intolerance did so at 6 or 12 months after MTX start. Consequently, the outcome for the prediction model was defined as MTX intolerance at 6 or 12 months after MTX start.

For the construction of the prediction model, patients with a completed MISS at 6 or 12 months were re-selected from the eligible cohort of 167 patients, resulting in 152 included patients (Figure 1).

### Potential clinical and genetic predictors

Potential clinical predictors (demographics, JIA category, disease characteristics, disease activity and biochemical measurements) were identified at baseline (Table 1). Potential genetic predictors were SNPs involved in the MTX metabolic pathways, with a high polymorphic allele frequency and documented functional effects [31]. SNPs were determined in the following genes: methylenetetrahydrofolate reductase (*MTHFR*), reduced folate carrier *(RFC)*, methionine synthase reductase *(MTRR)*, inosine triphosphatase *(ITPA)*, adenosine monophosphate deaminase *(AMPD)*, aminoimidazole-4-carboxamide ribonucleotide transformylase *(ATIC)*, adenosine-deaminase *(ADA)*, adenosine A2A receptor *(ADORA2A)*, multidrug resistance (*MDR)* 1, multidrug resistance protein *(MRP)* 1*–*5, breast cancer resistance protein *(BCRP)*, folylpolyglutamate synthase *(FPGS)*, gamma glutamyl hydrolase *(GGH)* and proton-coupled folate transporter *(PCFT)* (Table 1).

### Statistical analysis

#### Prediction model construction

The prediction model was constructed in several steps. First, missing values were imputed using multivariate imputation by chained equations (MICE) [32]. This was done to ensure that all collected data could be used for the development of the model. Second, to facilitate implementation of the model in daily clinical practice, continuous variables were dichotomised or categorised, according to patterns in the data or the risk gradients across percentiles, and the cut-off points with the lowest p-value on the log-likelihood ratio test (i.e. those yielding the optimal association) were chosen [33]. Third, all variables were entered in a univariable logistic regression analysis. The results are presented as regression coefficients (β) and odds ratios (OR) with 95% confidence intervals (95% CI). The regression coefficients are an indication of the direction and the magnitude of the effect of the individual predictors, whereas the ORs with 95% CI indicate the significance of the association.

Variables with a p-value <0.20 on the log-likelihood ratio test in the univariable analysis were eligible for inclusion in the multivariable logistic regression analysis. The maximum number of included variables equalled the square root of the number of cases (MTX intolerant patients) in the cohort. If more variables were eligible than the allowed maximum, or if variables correlated (Spearman’s |rho| >0.40), those with the lowest p-value on the log-likelihood ratio test were included in the multivariable analysis. In addition, presence of effect modification by the predictors in the model was assessed. Effect modification is the situation in which the effect of one predictor on the outcome is modified by the value of another factor. For example, the effect of a predictor may differ between boys and girls. Statistically, this is tested by adding interaction terms to the model, allowing the regression coefficients to take different values for different categories of patients.

Predictive power of the model was assessed with the C-statistic, which reflects the percentage of patients classified correctly. To determine whether the model fit the data well, the Hosmer-Lemeshow test was employed. Multicollinearity was tested with variance inflation factors (VIF).

#### Prediction model validation and risk score computation

All prediction models need to be validated. Since no independent cohort was available, the model was internally validated using an established statistical technique, called bootstrap [34-36]. In short, 200 bootstrap cohorts (of equal size as the original dataset, n = 152) were randomly drawn, with replacement, from the cases in the original dataset. Next, to each bootstrap cohort, bootstrap multivariable models were fitted (200 in total) using exactly the same methods as described above for the original model, and the corresponding C-statistics (C_boot_) were determined. Then, the probability of MTX intolerance of the patients in the original dataset was calculated using each of these multivariable models, resulting in another set of C-statistics (C_boot-original_) reflecting the percentage of patients predicted correctly according to each of these models. The difference between C_boot-original_ values and C_boot_ values is an estimate of the so-called optimism value (i.e. how much the original model fitted to the original dataset was optimistic compared to the “real” performance of the model in the population). Therefore, in order to obtain the final adjusted C-statistic, indicating the “real” performance of the model in the population, [36] two additional steps need to be performed: a) Subtraction of C_boot-original_ values from C_boot_ values and averaging them in order to obtain the optimism value; b) Subtraction of this optimism value from C_original_ (the C-statistic of the original model, developed in the original dataset), thus obtaining the final adjusted C-statistic. Furthermore, to correct for overfitting, the regression coefficients were reduced with a shrinkage factor, calculated from the bootstrap re-sampling.

All the above mentioned procedures were performed twice. Firstly, only the routinely available clinical variables were considered as potential predictors. Secondly, SNPs were also considered as potential predictors in order to determine whether they contributed to the prediction of MTX intolerance.

To compute a risk score of becoming MTX intolerant, the shrunken regression coefficients were multiplied and rounded off to obtain simple scores that sum up to a total risk score. Sensitivity, specificity, positive predictive value (PPV), negative predictive value (NPV) and accuracy of various cut-off points were calculated.

Statistical analyses were carried out with R statistics version 2.15.0 (R Foundation for Statistical Computing, Vienna, Austria), using the packages Hmisc (by Frank E Harrell Jr with contributions from many other users, version 3.9-3, 2012) and mice [32].

## Results

### Baseline characteristics of the prediction model cohort

The prediction model was constructed in 152 patients. According to the outcome as defined above, 51 (33.6%) patients were MTX intolerant (Table 2). Intolerant and tolerant patients did not differ regarding the proportion of MTX re-starters, MTX dose, route of administration, concomitant medication use or disease activity (Juvenile Arthritis Disease Activity score [JADAS-27]) at 6 and 12 months after MTX start (data not shown).

Nineteen (12.5%) patients discontinued MTX treatment during the follow-up, because of MTX intolerance (n = 8), disease remission (n = 3), insufficient effect (n = 2), MTX toxicity (increased liver enzymes: n = 1) or other reasons (n = 5). Patients also switched the route of administration due to gastrointestinal complaints (either from oral to subcutaneous or vice versa): 8 patients after 3 months, 6 patients after 6 months and 1 patient after 12 months.

Baseline characteristics are depicted in Table 1. Thirty-one patients (20.4%) had re-started MTX treatment due to a relapse after at least three months discontinuation. The majority of patients had either oligoarticular or polyarticular JIA (82.9%), with high disease activity (median JADAS-27 of 12.7 [interquartile range 7.6-18.2]). Median MTX dose was 9.9 mg/m^2^/week, administered mostly as oral MTX (97.4%) with concomitant use of folic acid (98.7%).

### Clinical prediction model

First, a model was constructed, containing clinical variables only, excluding the SNPs. Ten clinical variables were associated in the univariable analysis with MTX intolerance (p < 0.20; Table 1). The maximum number of variables allowed in the multivariable analysis was seven. Those with the lowest p-value were selected for the clinical prediction model, namely JIA category, JADAS-27, parent/patient assessment of pain, antinuclear antibody (ANA), alanine aminotransferase (ALT), thrombocytes and creatinine, and an interaction term between creatinine and JIA category was added. The C-statistic of the clinical prediction model was 77.5% (Table 3). The model fit the data well, as shown by a non-significant Hosmer-Lemeshow test (p = 0.705). There was no multicollinearity (data not shown).

**Table 3:** **Prediction model and scores for MTX intolerance**

**Predictors**		**OR (95%-CI)**	**p-value**	**β** ^**a**^	**Score** ^**b**^
JIA category					
Oligoarticular (persistent/extended)		Reference			0
Polyarticular (RF negative/positive)		4.99 (1.36-18.34)	0.016	0.914	5
Other (systemic/psoriatic/enthesitis)		0.93 (0.16-5.49)	0.935	−0.042	0
ANA	Positive	1.98 (0.83-4.68)	0.122	0.387	2
Parent/patient assessment of pain	≤3 cm	Reference			0
	3-6 cm	2.06 (0.72-5.89)	0.175	0.412	2
	>6 cm	0.60 (0.17-2.07)	0.421	−0.288	−1
JADAS-27	≤5	Reference			0
	5-15	0.35 (0.08-1.56)	0.168	−0.599	−3
	>15	0.77 (0.14-4.32)	0.766	−0.150	−1
Thrombocytes	>350 × 10^9^/L	1.27 (0.49-3.27)	0.621	0.136	1
ALT	>12 IU/L	0.39 (0.16-0.96)	0.040	−0.534	−3
Creatinine	>50 μmol/L	1.37 (0.33-5.67)	0.665	0.179	1
Interaction term creatinine: JIA category					
>50 μmol/L & polyarticular arthritis		0.17 (0.02-1.35)	0.093	−1.022	−5
>50 μmol/L & other JIA category		0.82 (0.07-9.74)	0.878	−0.110	−1
Constant				−0.039	7
C-statistic		77.5%			
C-statistic (optimism-corrected by bootstrap)		66.7%			
Hosmer-Lemeshow test (p-value)		0.705			

***Abbreviations:*** ALT, alanine aminotransferase; ANA, anti-nuclear antibody; CI, confidence interval; JADAS, juvenile arthritis disease activity score; JIA, juvenile idiopathic arthritis; MTX, methotrexate; OR, odds ratio; RF, rheumatoid factor.

^**a**^These are shrunk coefficients (by factor 0.5688) to correct for overfitting.

^**b**^Shrunk coefficients were multiplied by 5 and rounded off to the nearest integer. The constant was adjusted to obtain the minimum score of 0.

### Clinical-genetic prediction model

Next, SNPs were considered as potential predictors in order to determine their contribution to MTX intolerance prediction. Four SNPs in the *MTRR, RFC, MDR-1* and *MRP-3* genes had univariable p-values of <0.20, however these p-values (range: 0.123-0.194) were generally higher than those of the clinical model variables (range: 0.048-0.161) (Table 1). Hence, since seven variables with the smallest p-values were selected for multivariable analysis, only the *MTRR* rs1801394 SNP, next to six clinical variables (those from the abovementioned clinical model, excluding thrombocytes), were included in the model. The model’s C-statistic was 77.7%.

### Prediction model validation

Both the clinical and the clinical-genetic prediction model were internally validated using bootstrapping. Upon internal validation, the corrected C-statistic of the clinical model was 66.7%, whereas the corrected C-statistic of the clinical-genetic model was 64.6%.

Since the clinical-genetic model did not perform better than the model with clinical variables, the latter was given preference as clinical variables are readily available at MTX start, making it easier to apply the model in clinical practice.

### Risk score

To enable health care professionals to use the model easily, the shrunken regression coefficients of the clinical model’s predictors, transformed into simple scores, were used to compute an individual risk score for being MTX intolerant. This score ranged from 0 to 17 points, with a higher score reflecting a higher probability of MTX intolerance (Table 3). The lowest predicted risk of being MTX intolerant was 18.9%, if the following predictors were present: oligoarticular JIA, negative ANA, parent/patient assessment of pain >6 cm, JADAS-27 of 5–15 points, thrombocytes ≤350 × 10^9^/L, ALT >12 IU/L and creatinine ≤50 μmol/L. The combination of these predictors resulted in a score of 0 [7 (the constant) + 0 + 0 + (−1) + (−3) + 0 + (−3) + 0] (Table 3). On the other hand, the highest predicted risk of being MTX intolerant was 85.9%, if the following predictors were present: polyarticular JIA, positive ANA, parent/patient assessment of pain of 3–6 cm, JADAS-27 ≤ 5 points, thrombocytes >350 × 10^9^/L, ALT ≤12 IU/L and creatinine ≤50 μmol/L. The combination of these predictors resulted in a score of 17 [7 + 5 + 2 + 2 + 0 + 1 + 0 + 0].

Within the 0–17 range, the diagnostic accuracy of different cut-off scores for predicting the risk of being MTX intolerant was evaluated by computing the corresponding sensitivity, specificity, PPV, NPV, and accuracy (Table 4). Our goal was to correctly identify as many future MTX intolerant patients as possible (high sensitivity), while attempting to avoid misidentification of tolerant patients as intolerant patients (moderate specificity). This was reached at the cut-off score ≥6, where 82% of intolerant patients and 56.1% of tolerant patients were identified correctly.

**Table 4:** **Diagnostic parameters of the risk score for various cut-off scores**

**Cut-off**	**Sensitivity (%)**	**Specificity (%)**	**PPV (%)**	**NPV (%)**	**Accuracy (%)**
≥4	93.4	29.9	50.3	85.7	57.3
≥5	87.8	46.1	55.3	83.3	64.1
≥6	82.0	56.1	58.7	80.4	67.3
≥7	69.2	69.9	63.6	74.9	69.6
≥8	58.7	80.3	69.4	71.9	71.0
≥9	46.0	86.8	72.6	67.9	69.2

***Abbreviations:*** NPV, negative predictive value; PPV, positive predictive value.

## Discussion

We developed and internally validated a prediction model for MTX intolerance at 6 or 12 months after MTX start in a large JIA cohort, consisting of routine clinical variables: JIA category, JADAS-27, parent/patient assessment of pain, ANA, ALT, thrombocytes, creatinine and an interaction term between creatinine and JIA category. The model classified 77.5% of patients correctly, and 66.7% after internal validation. It should be validated in an independent cohort and updated with other predictors.

In our model, patients who had more pain (>6 cm), higher baseline disease activity assessed with JADAS-27 and higher ALT, had a lower risk to become MTX intolerant. On the other hand, patients with positive ANA, who had less pain (3–6 cm), higher thrombocyte levels and higher creatinine, had an increased risk of MTX intolerance. Creatinine level and age were correlated, so creatinin can be regarded as a surrogate marker for age (median age was 7.5 years [patients with creatinine ≤50 μmol/L] versus 13.7 years [creatinine >50 μmol/L]). The relationship between JIA category, creatinine (age) and MTX intolerance was complex: In younger patients, polyarticular JIA was a strong predictor for intolerance (score 5, Table 3), whereas in older patients this effect disappeared (score 5 for polyarticular JIA and −5 for the interaction term between older patients (higher creatinine) and polyarticular JIA).

To predict which patients are prone to develop MTX intolerance, our risk score could be readily used by clinicians, since it is based on clinical variables, which are routinely determined and available for all JIA patients before MTX start. At the cut-off score of ≥6, as many as 82% of intolerant patients were classified correctly (high sensitivity), while maintaining correct classification of 56.1% of tolerant patients (modest specificity). Table 4 provides the sensitivity and specificity of other potential cut off points.

Identification of patients at risk increases patients’ and clinicians’ awareness of MTX intolerance. In patients at risk, clinicians should frequently (i.e. every 4 weeks) monitor MTX-related gastrointestinal adverse effects, using the MISS, from the very start of MTX treatment. This would enable clinicians to treat the emerging physical symptoms early, for example by lowering MTX dose, [4] adding anti-emetics [18] or applying behavioural therapy, [5] thus preventing the development of a classical conditioning response [15] and hence MTX intolerance. The effect of these timely interventions on the development of MTX intolerance should be determined in a clinical trial.

The outcome of our prediction model was defined as MTX intolerance at 6 or 12 months after MTX start, since the majority of patients developing MTX intolerance did so at these time-points. The later onset of MTX intolerance is consistent with the notion that the development of MTX intolerance is governed by a classical conditioning response, which worsens over time [5,14]. Moreover, in our previous cross-sectional study in patients with longer MTX use (interquartile range: 0.6-3.6 years), we demonstrated higher prevalence of MTX intolerance (50.5-67.5%) compared to the prevalence of 34.1% in the present longitudinal study during the first year of MTX treatment [14]. This also supports the notion that MTX intolerance takes time to develop and that longer MTX use may increase the risk of MTX intolerance. To determine whether the risk of MTX intolerance indeed increases with longer MTX use, development of MTX intolerance should be monitored beyond one year of MTX use. Nevertheless, MTX intolerance ensued in 15.8% of patients already after 3 months of MTX use. Interestingly, patients who had restarted MTX had a higher risk of becoming intolerant after 3 months than those newly starting MTX (36% versus 12.7%, p = 0.015).

To our knowledge, no previous studies have developed a similar model and a corresponding risk score to predict the occurrence of MTX-induced gastrointestinal adverse effects in JIA. In a recently published paper, predictors for MTX adverse events in JIA patients, including the predictors in the current model, were reviewed. Only a few candidate predictors were elucidated, and validation of these lacked [29].

Our study did not identify genotypes as predictors for intolerance. In contrast, in RA, two studies identified combinations of risk genotypes to predict adverse effects in general and gastrointestinal adverse effects in particular [20,26]. In our study, only 4 of 27 SNPs were moderately associated with MTX intolerance and only one SNP could be included in the clinical-genetic model, which had comparable predictive power as the clinical model. Previously, in RA and JIA, significant associations (p < 0.05) were reported between SNPs in the *MTHFR, ATIC, ADORA, MRP2/ABCC2* and *GGH* genes and gastrointestinal adverse effects [19-22,24-28,37,38]. SNPs in these genes were not associated with MTX intolerance in our study, which could be due to disparities in patient groups (RA versus JIA), cohorts (cross-sectional versus longitudinal), and the definition of MTX-induced gastrointestinal complaints (after MTX versus before *and* after MTX use). These results taken together with our current study show that it is still difficult to predict reliably the risk of developing MTX adverse events in general and MTX intolerance in particular.

The strengths of our study were that MTX intolerance was assessed using a validated questionnaire. In addition, the model was constructed and internally validated in a large prospective JIA cohort. Internal validation using bootstrapping is an established method to estimate the performance of a prediction model in the population, comparable to external validation in an independent cohort [34-36].

## Conclusions

In conclusion, we developed and internally validated a clinical prediction model for MTX intolerance in a large JIA cohort. It is an easy-to-use tool to identify patients at risk of developing MTX intolerance, and in turn to monitor them closely and intervene timely, in order to prevent MTX intolerance and its negative impact on patients’ daily lives, compliance and continuation of an effective treatment. In its current composition, the model performs moderately well and should be validated in an independent cohort and updated with new predictor variables before it can be broadly used in clinical practice.
